# Hand washing with soap and water together with behavioural recommendations prevents infections in common work environment: an open cluster-randomized trial

**DOI:** 10.1186/1745-6215-13-10

**Published:** 2012-01-16

**Authors:** Carita Savolainen-Kopra, Jaason Haapakoski, Piia A Peltola, Thedi Ziegler, Terttu Korpela, Pirjo Anttila, Ali Amiryousefi, Pentti Huovinen, Markku Huvinen, Heikki Noronen, Pia Riikkala, Merja Roivainen, Petri Ruutu, Juha Teirilä, Erkki Vartiainen, Tapani Hovi

**Affiliations:** 1 National Institute for Health and Welfare (THL), Department of Infectious Disease Surveillance and Control, Intestinal Viruses Unit, P.O. Box 30, FIN-00271 Helsinki, Finland; 2 National Institute for Health and Welfare (THL), Information Department, Information Systems Development and Support Unit, Helsinki, Finland; 3 National Institute for Health and Welfare (THL), Department of Vaccination and Immune Protection, Vaccine Research Unit, Helsinki, Finland; 4 National Institute for Health and Welfare (THL), Department of Vaccination and Immune Protection, Viral Infections Unit, Helsinki, Finland; 5 Kesko Oyj, Helsinki, Finland; 6 University of Helsinki, Department of Mathematics and Statistics; 7 National Institute for Health and Welfare (THL), Division of Health Protection and Department of Medical Microbiology and Immunology, University of Turku, Turku, Finland; 8 Outokumpu Oyj and Outotec Oyj, Espoo, Finland; 9 Nordea Bank Finland Plc, Helsinki, Finland; 10 National Institute for Health and Welfare (THL), Department of Infectious Disease Surveillance and Control; 11 SOK, Helsinki, Finland; 12 National Institute for Health and Welfare (THL), Division of Welfare and Health Promotion

## Abstract

**Background:**

Hand hygiene is considered as an important means of infection control. We explored whether guided hand hygiene together with transmission-limiting behaviour reduces infection episodes and lost days of work in a common work environment in an open cluster-randomized 3-arm intervention trial.

**Methods:**

A total of 21 clusters (683 persons) were randomized to implement hand hygiene with soap and water (257 persons), with alcohol-based hand rub (202 persons), or to serve as a control (224 persons). Participants in both intervention arms also received standardized instructions on how to limit the transmission of infections. The intervention period (16 months) included the emergence of the 2009 influenza pandemic and the subsequent national hand hygiene campaign influencing also the control arm.

**Results:**

In the total follow-up period there was a 6.7% reduction of infection episodes in the soap-and water arm (p = 0.04). Before the onset of the anti-pandemic campaign, a statistically significant (p = 0.002) difference in the mean occurrence of infection episodes was observed between the control (6.0 per year) and the soap-and-water arm (5.0 per year) but not between the control and the alcohol-rub arm (5.6 per year). Neither intervention had a decreasing effect on absence from work.

**Conclusions:**

We conclude that intensified hand hygiene using water and soap together with behavioural recommendations can reduce the occurrence of self-reported acute illnesses in common work environment. Surprisingly, the occurrence of reported sick leaves also increased in the soap-and water-arm.

**Trial Registration:**

ClinicalTrials.gov: NCT00981877

**Source of funding:**

The Finnish Work Environment Fund and the National Institute for Health and Welfare.

## Background

Enhanced hand hygiene is a well established means to prevent the transmission of infections in hospital settings [1] as well as in other semi-closed environments with high infection pressure, such as day care centers [2-5], schools [6,7] and military service [8].

Enhanced hand hygiene has been studied as a means to prevent the transmission of respiratory and diarrheal infections in community settings. In recent meta-analyses, however, the overall evidence has been considered inconclusive due to differences in study designs and difficulty in adjusting for confounding factors [9,10]. A study published during the preparation of this manuscript reported a reduction in infection episodes through the use of alcohol-based hand disinfectants in an office work place [11].

Data on the effect of hand hygiene on the transmission of influenza would be important for strengthening the evidence base for current recommendations to prevent seasonal and pandemic influenza. In a recent study, enhanced hand hygiene together with the use of a surgical face mask prevented influenza virus transmission within households when implemented within 36 hours of the onset of symptoms in the index patient [12]. In contrast, influenza transmission was not reduced by interventions to promote hand washing and face mask use in another study [13].

We studied whether enhanced hand hygiene together with behavioural recommendations aimed at reducing transmission by droplets during coughing or sneezing could reduce infection episodes and absence from work in a common office work environment. In our cluster-randomized trial, we compared a non-intervention group to traditional hand-washing with soap and water and to alcohol-based hand rubbing gel, combined with advice on coughing or sneezing behaviour for both intervention groups. Randomization in office worker clusters was used instead of personal randomization for two main reasons: first, members of a given office work unit can be considered to form a single circulation environment for infectious agents and, secondly, all participants in such a cluster should implement uniform hand hygiene habits so as to achieve optimal adherence to intended behaviour.

## Methods

### Study design

The efficacy of enhanced hand hygiene on infection episodes and absences from work in office environments was studied in an open, cluster-randomized intervention trial. A detailed description of the study design has been reported earlier [14]. The protocol was accepted by the Institutional Review Board (reference number 9/2008).

**Figure 1:**
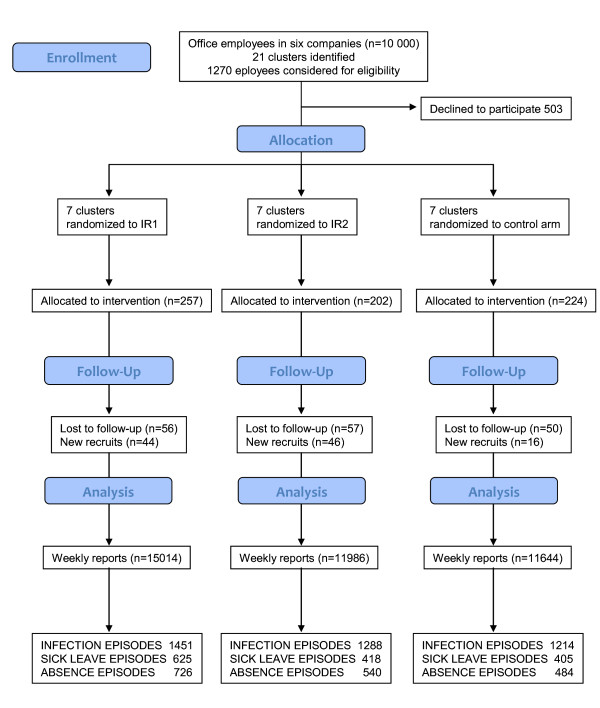
**Trial design, recruitment and reporting**.

Briefly, a total of 21 distinct office work units in six corporations in the Helsinki Region were enrolled in the study in collaboration with the occupational health clinics serving these corporations [14]. Altogether the corporations employed some 10 000 staff (Figure 1). An electronic contagion risk survey questionnaire was sent to all employees of the 21 target units. When taking part in the survey questionnaire, the respondents were asked their willingness to participate in the intervention study. An arbitrary virus transmission risk score for each cluster was calculated. Living with preschool-aged children attending day care had the highest weight in the risk calculation [14]. Based on the score, matching and randomization of the clusters in the three trial arms was performed as described in detail elsewhere [14].

The trial arms were: IR1, the soap and water wash arm; IR2, the alcohol-based hand rub arm; and the control arm (C). Toilets at the work units were equipped with liquid hand soap (all arms) or alcohol-based hand rub (IR2). Participants also obtained bottles of hand hygiene product to be used at home and, in the case of alcohol hand rub, personally in the office. In addition to personal guidance in hand cleansing specific to each arm, the participants of IR1 and IR2 received guidance on how to otherwise limit the transmission of infections, e.g. coughing, sneezing into a disposable handkerchief or alternatively the sleeve, and avoiding shaking hands [14]. Participants of the control arm did not receive any guidance regarding hand hygiene or limiting transmission of infections. The interventions were not blinded to any party involved (i.e. the study group, participants or the occupational health services).

Daily infection symptoms, sick leave and absences from work for any reason were recorded by weekly self-report using an internet-based questionnaire, a link to which was sent via email [14]. Symptoms typical of acute respiratory (RTI) or gastrointestinal infection (GTI) were described in detail during the advance training and repeated in the weekly email. The software used for data collection was acquired from Digium Enterprises, Espoo, Finland. The data were stored in an in-house database for monitoring and analyses.

### Monitoring of implementation of the intervention

Adherence to the assigned intervention was assessed by an electronic survey of transmission limiting habits, as described in detail elsewhere [14]. In the intervention clusters, the use of soap (IR1) and alcohol-based disinfectant (IR2) provided to the participants specifically for personal use was recorded. The study nurse regularly visited the intervention clusters throughout the intervention period, checked the availability of soap and alcohol rub and assisted in any practical problems. The liquid soap distributed was "Erisan Nonsid" (Farmos Inc., Turku, Finland). This was also the only soap available in the toilets used by the study clusters. An 80% ethanol-containing disinfecting hand rub "LV" (Berner Inc., Helsinki, Finland) was used in IR2. The products used are market leaders of their type in Finland.

### Surveillance for viral infections

Between November 2008 and May 2010, the seven occupational health clinics serving the six participating corporations were advised to collect, using standard techniques, two to three respiratory samples per week from typical RTI patients and also faecal samples from a few representative patients with gastrointestinal symptoms when a GTI outbreak was suspected. The samples could originate from the study participants and also from work units not included in the study. In the laboratory, viral nucleic acids were extracted with well characterized commercial kits and tested by validated real-time PCR methods to detect influenza A and B viruses, respiratory syncytial virus, parainfluenza virus types 1, 2, and 3, adenoviruses, human rhinoviruses and human enteroviruses from respiratory specimens, and norovirus from faecal specimens (detailed descriptions of the test procedures are available from the authors).

### Primary endpoints and outcome measures

The predefined primary endpoints were (1) the number of reported infection episodes in a cluster per total reported weeks; and (2) the number of reported sick leave episodes in a cluster per total reported weeks.

### Data management and rationale for analysis

Successive weekly reports from a given participant were combined into a single continuum of daily records. Disease episodes were defined as the number of successive symptomatic days, allowing one intervening asymptomatic day. Sick leave episodes were restricted to days off due to RTI or GTI of the study subject, while absence episodes also included days off due to RTI or GTI of a dependent [14]. Occurrence of episodes was expressed as the proportion of total reported person weeks in a cluster or trial arm with a recorded onset of the indicated episode, resulting in "infection proportion", "RTI proportion", "GTI proportion", "sick leave proportion", and "absence proportion". The mean number of episodes per person-year was obtained by multiplying the respective proportion by 52.

The designated infection risk score of the clusters used in randomization and for matching the arms [14] did not correlate with the observed occurrence of infections. Therefore, the triplets of similar clusters were ignored in the statistical analysis, and instead, datasets derived from the total arms were used.

### Statistical analysis

According to the null hypothesis none of the above proportions in the IR1 or in the IR2 was different from the corresponding proportion in the control arm. This hypothesis was tested separately for each proportion using the Yate's Chi- Square (prop.test) with the R-statistical package http://www.r-project.org/. The binary variable was formulated and made the proportion of reported weeks with an onset of defined episode in each arm. The equality of each of these proportions for the soap and alcohol intervention arms with the corresponding proportion obtained for the control arm was tested separately with Yate's Chi- Square test (Pearson's Chi-square with continuity correction). The p-values given represent the probability that random sampling would lead to a difference between sample proportions.

The unexpected influenza A/H1N1 pandemic in Finland in summer and fall 2009 resulted in a nationwide campaign for improved hand hygiene from August, 2009, onwards. Therefore, the analyses were also performed separately for the period before the end of July, 2009 (25 weeks; "before the pandemic"), and thereafter until the end of May, 2010 (43 weeks; "during and after the pandemic"). Similarly, proportion test was exploited in the analysis of the significance of differences between the arms and between different time points concerning the answers to the questions in the survey on transmission-limiting habits.

## Results

### Recruitment

Recruitment took place in January and February 2009. Altogether 683 persons volunteered to participate in the study. Characteristics of the participants are shown in Table 1. The interventions lasted for 15-16 months, until the end of May 2010. The percentage of staff in each cluster participating in the study ranged from 12-51%, with a mean of 32.7% and a median of 32.5% (Table 2).

**Table 1:** Characteristics of the participants in different intervention arms.

INTERVENTION ARM	N OF INITIAL PARTICIPANTS	AGE RANGE (STD)	MEAN AGE	PROPORTION OF THOSE WITH CHILDREN IN DAY CARE	MEAN OF RELATIVE RISK SUM
IR 1	257	22-64 (10.2)	45.1	0.118	63.9
IR2	202	20-63 (10.1)	42.7	0.128	55.0
CONTROL	224	21-62 (11.1)	42.8	0.144	59.9

**Table 2:** Cluster characteristics, and outcome data from the total study period, weeks 7-74 (a total of 68 weeks), as well as from the first part of the study prior to the influenza A/H1N1 pandemic for the infection proportion (no. of infection episodes/reported weeks).

Random. set and triplet	Arm and cluster code	Total staff number	Relative infection risk sum	**Initial number of particip**.	Soap or disinfectant usage per participant	Number of reported weeks	Median coverage% of weeks	No. of infection episodes	Infection proportion, whole study	Infection proportion, before the pandemic	Sick leave proportion	Absence proportion
I/A	IR2 12	160	35	52	5.3	2997	89.5	305	0.102	0.113	0.037	0.040
	IR1 16	100	39	48	7.5	2515	89.7	195	0.078	0.095	0.029	0.030
	C 26	80	45	22	nr	1159	88.9	111	0.096	0.096	0.054	0.057
I/B	C 15	100	45	36	nr	1740	73.6	122	0.070	0.083	0.026	0.037
	IR2 14	100	47	12	15.8	648	76.9	72	0.111	0.111	0.045	0.057
	IR1 13	160	50	57	9.2	3544	83.1	443	0.125	0.115	0.072	0.078
I/C	IR2 24	76	51	25	8.1	1537	89.1	263	0.171	0.170	0.034	0.051
	IR1 20	50	55	12	2.8	645	91.7	55	0.085	0.110	0.029	0.029
	C 25	96	57	27	nr	1248	73.9	174	0.139	0.185	0.036	0.043
I/D	IR2 21	50	57	16	7.5	1132	86.4	102	0.090	0.087	0.027	0.034
	C 22	50	65	20	nr	1174	94.7	133	0.113	0.135	0.035	0.040
	IR1 18	82	72	31	5.1	1807	96.3	131	0.072	0.061	0.031	0.032
II/A	IR2 6	118	41	37	5.4	2078	86.1	213	0.103	0.096	0.037	0.045
	C 3	150	50	50	nr	2690	89.2	313	0.116	0.127	0.031	0.037
	IR1 10	123	58	54	3.9	3167	91.7	312	0.099	0.094	0.041	0.055
II/B	IR2 5	110	68	33	7.1	2079	97.1	194	0.093	0.080	0.031	0.047
	IR1 7	45	69	23	6.2	1370	91.3	105	0.077	0.075	0.034	0.039
	C 2	150	70	33	nr	1841	88.1	184	0.100	0.095	0.037	0.043
II/C	IR2 9	100	86	27	6.3	1515	85.2	139	0.092	0.087	0.035	0.050
	C 8	160	87	36	nr	1792	82.4	177	0.099	0.096	0.033	0.041
	IR1 4	80	104	32	1.3	1966	91.3	210	0.107	0.105	0.024	0.034
Total	IR1	640	903	257	6.1	15014	89.5	1451	0.097	**0.096**	0.037	0.043
	IR2	714	774	202	6.9	11986	89.0	1288	0.107	**0.108**	0.035	0.046
	C	786	851	224	nr	11644	83.8	1214	0.104	**0.115**	0.036	0.043

Totals in the three last lines were calculated from the cluster means.

### Drop-outs, new recruiting and reporting coverage during follow-up

Seventy six percent of volunteers who started reporting continued to do so until the end of the study (Figure 1). The most common reason for discontinuing reporting was quitting working in the study cluster. Because of new recruiting in most clusters, the total number of reporting participants at the end of the trial was 626 or 91.7% compared to that at the beginning.

**Figure 2:**
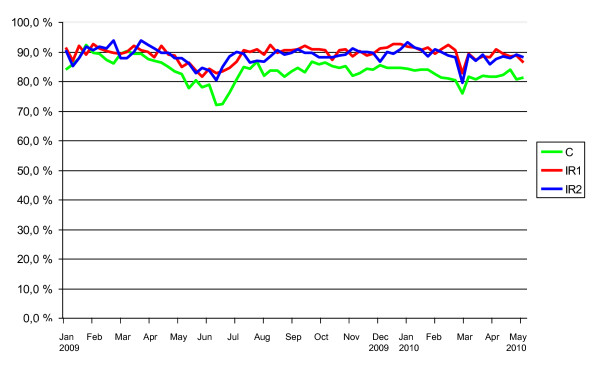
**Reporting coverage in the study arms**.

The proportion of weekly reports received from participants during the follow-up was generally very high, and similar in the three study arms throughout the study (Figure 2). An automated email reminder to the participant was sent if a weekly report was not received within five days unless the participant had informed in advance that she/he will be on vacation or on business travel. Participants were given the opportunity to report on events during the vacation. Altogether 38 644 weekly reports were received.

### Main outcome measures

In the total follow-up period there was a 6.7% reduction of infection episodes in the IR1 (p = 0.04). In the subanalysis of data collected before the influenza A/H1N1 pandemic there were statistically significantly fewer infection episodes in IR1 (proportion 0.096 corresponding to 5.0 per person year) than in the control arm (proportion 0.115 corresponding to 6.0 per person year) equating to a reduction of 16.7% (Table 3). A statistically significant reduction was observed in the infection episodes of the control arm when periods before and after onset of the pandemic were compared (0.115 vs. 0.098) (Table 3). When broken down to RTI and GTI episodes, IR1 had the lowest number in both categories over the entire study period. The difference to control was statistically significant in GTI episodes (p = 0.03). No reduction in sick leave or absence episodes was observed prior to the pandemic. Rather, during and after the pandemic a statistically significant increase was seen in IR1.

**Table 3:** Outcome measures in different study periods.

OUTCOME MEASURE	INTERVENTION ARM	BEFORE THE PANDEMIC	DURING AND AFTER THE PANDEMIC	TOTAL FOLLOW-UP TIME
**INFECTION EPISODES/REPORT WEEKS**	IR1	**0.096 (p = 0.002 vs. C)**	0.097	0.097 (p = 0.04 vs. C)
	IR2	0.108	0.107	0.107
	CONTROL	0.115	**0.098 (p = 0.005 vs. before pandemic)**	0.104
**RESPIRATORY INFECTION EPISODES/REPORT WEEKS**	IR1	**0.074 (p = 0.01 vs. C)**	0.078	0.076
	IR2	0.084	0.085 (p = 0.03 vs. C)	0.085
	CONTROL	0.088	0.075	0.080
**GASTROINTESSINAL INFECTION EPISODES/REPORT WEEKS**	IR1	0.015	0.011	**0.012 (p = 0.03 vs. C)**
	IR2	0.017	0.015	0.016
	CONTROL	0.018	0.015	0.016
**SICK LEAVE EPISODES/REPORT WEEKS**	IR1	0.031	0.047	0.042
	IR2	0.027	0.039	0.035
	CONTROL	0.030	0.038 (p = 0.003 vs. IR1)	0.035 (p = 0.004 vs. IR1)
**ABSENCE EPISODES/REPORT WEEKS**	IR1	0.038	0.054	0.048
	IR2	0.036	0.050	0.045
	CONTROL	0.038	0.044 (p = 0.003 vs. IR1)	0.042 (p = 0.009 vs. IR1)

Numbers represent proportions calculated from the number of reported episodes in a trial arm per total reported weeks. Proportions can be converted to episodes per person year by multiplying by 52. Statistically significant differences indicating efficacy of the intervention are shown in bold face.

### Monthly distribution of infection episodes

**Figure 3:**
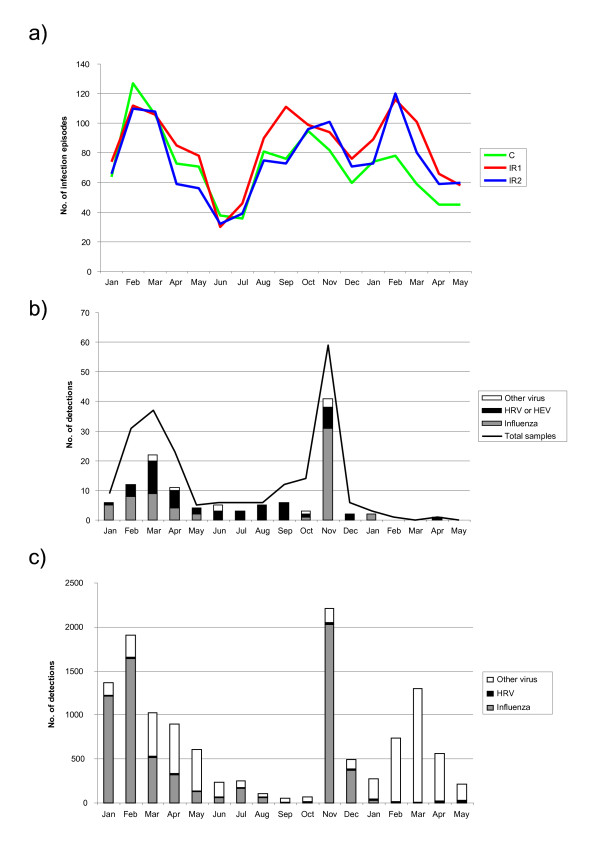
**a) Temporal distribution of all infection episodes/weekly reports in different intervention arms during the study**. C, control arm; IR1, soap and water using arm; IR2, alcohol-based hand rub using arm. b) Respiratory viruses detected in the study between January 2009-May 2010. Influenza detections during November-December 2009 consist of pandemic influenza A/H1N1. Other viruses includes adenovirus (N = 1), respiratory syncytial virus (N = 1) and parainfluenzaviruses (N = 7), c) Respiratory virus findings reported to the National Infectious Disease Registry in Finland. Other virus includes adenovirus (N = 872), respiratory syncytial virus (N = 4048) and parainfluenzaviruses (N = 608).

The monthly distribution of the proportion of all infection episodes/weekly reports showed an expected seasonal variation in all three arms (Figure 3a). The A/H1N1 pandemic did not cause a major peak in reported infection episodes, but in viral surveillance there was a peak for A/H1N1 in November 2009 (Figures 3b and 3c). All respiratory viruses under surveillance were detected among 219 specimens from patients visiting the occupational health clinics of the participating corporations (Figure 3b). Human rhinovirus was the most frequently detected pathogen (23.2%), followed by influenza A/H1N1 (15.6%), influenza A untyped (8.9%) and influenza B (4.5%). Parainfluenzaviruses 1, 2 and 3, respiratory syncytial virus and adenovirus were also detected. The epidemic peaks coincided with those notified by clinical laboratories to the National Infectious Disease Registry http://www.thl.fi/ttr (Figure 3c) and reported infection episodes in this study (Figure 3a). During winter/spring 2009, at the peak period for infection episodes, many different viruses were detected; i.e. no single outbreak explained the reported infection episodes. Only one of the 11 tested faecal specimens was positive for norovirus.

### Adherence to interventions

The recorded use of soap and alcohol-based disinfectant for personal use was smaller than the predicted use based on hand hygiene instructions (Table 2). The observed differences in soap or disinfectant use between study population clusters showed no correlation with the number of reported infection episodes.

**Figure 4:**
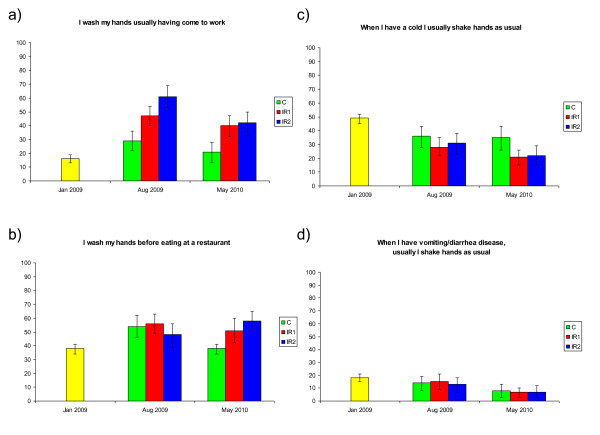
**Results of the survey of behavioral habits**. C, control arm; IR1, soap and water using arm; IR2, alcohol-based hand rub using arm. Results of January 2009 (yellow) represent baseline before any of the recruited office work employees had received guidance on hand hygiene.

The survey on transmission-limiting habits was carried out three times, before randomization, at the time of the intense influenza A/H1N1 pandemic media coverage in August 2009, and again at the end of the follow-up period in May 2010 (13). Some survey indicators are depicted in Figure 4. The high initial level of hand hygiene in several sectors improved in all arms, including the control arm (p = 0.0005 or less; Figure 4a and 4b). Avoiding shaking hands when ill with respiratory or gastrointestinal infection became more common during the progression of the study (Figure 4c and 4d) in all study groups, and remained high in both intervention arms (p≈0 for all measuring points as compared to the starting level).

## Discussion

This controlled intervention trial demonstrated a significant reduction in infection episodes, especially in respiratory infections, in the trial arm carrying out enhanced conventional hand washing with soap and water, combined with instructions on how to reduce transmission while coughing or sneezing. In contrast, hand rubbing with alcohol-based disinfectant, together with guidance on coughing or sneezing, did not reduce infection episodes compared with the control group. The influenza pandemic 2009 triggered an intense national hand washing campaign, during and after which no differences were seen between the two interventions and the control arm. This is partially explained by the observation that the occurrence of infections decreased significantly in the control arm.

Cluster effect on the distribution of individual end points is often taken in account in statistical analysis of the results of intervention trials. We compared the cumulative results of the two intervention arms with the control arm directly, ignoring possible cluster effects. Baseline occurrence of infection episodes among members of a cluster defined by a working team might be influenced by behavioural habits typical of the team influencing the transmission of infections, and in this type of trial, by cluster-specific intensity of applying instructed hand-cleaning procedures and behavioural change. However, successive infection episodes within a cluster are not a direct reflection of transmission of the infectious agent within the cluster but as likely, due to new introductions, i.e., infections contracted in non-work related contacts of individual study participants. Work-related cluster effect is unlikely to affect the occurrence of these latter infections while the incidence of community-based infections is known to vary rather randomly between different subpopulations and by time within a given subpopulation. Therefore, inclusion of the extra variability due to cluster-effect in statistical analysis might result in missing true differences in a set-up like ours where the number of clusters was rather limited for practical reasons [14].

Our main result of reduced respiratory infections through hand washing with soap and water combined with advice on coughing and sneezing is in agreement with several previously published studies in semi-closed populations such as hospitals and children's day care centres [15,16]. However, there are no data (6, 7) available on this intervention in adult populations in a regular office environment where contact patterns are likely to be different from the semi-closed environments. A decrease of 50% was reported in the incidence of childhood pneumonia following intensified hand washing with soap and water in Pakistan. The absence of protection from infection episodes in the alcohol hand rub intervention group is at variance with a recent publication on a similar intervention reporting a remarkable reduction of disease episodes [11] and with some earlier studies [17,18]. We do not know the reason for the discrepant results but one can speculate about the potential effects of putative differences in the disinfectant composition, varying pre-study levels of hand washing routines, and different study design. Also, we do not want to rule out possibility that alcohol hand rub would have a decreasing effect on infection episodes, if the number of follow-up persons was greater. On the other hand, the current result is in agreement with our unpublished observations on the capacity of a single round of instructed hand cleaning to remove infectious human rhinovirus administered on the skin of the back of the hand. Washing with soap and water appeared to be much more efficient than rubbing with the alcohol-based disinfectant (Savolainen-Kopra et al., unpublished observations). While participants in the control arm were not forced not to use soap and water or alcohol rub, the observed effect in the intervention cannot be due to behavioural recommendations only because the same recommendations were given to both intervention arms IR1 and IR2.

The interventions continued over two winter seasons in order to cover different seasonal virus epidemics. Structurally different viruses might differ in sensitivity to the hand washing procedures employed. However, the influenza pandemic with influenza A H1N1 2009 triggered an intense national hand washing campaign that compromised the implementation of our study, with the control clusters in our study also being exposed to the information in the public media, which was further tailored to all staff by the occupational health units in the participating corporations. Rather than stopping the trial prematurely, we decided to continue it through the planned period and analyze the results in two blocks of follow-up time, "before the pandemic" and "during and after the pandemic", respectively. Subsequently, a Eurobarometer survey in all EU countries showed that in Finland over 40% of the adult population reported to have changed their behaviour so as to improve their protection from influenza [19]. According to that survey, the change was almost exclusively seen in improved hand hygiene, with hardly any change in behaviour related to coughing or sneezing. During and after the pandemic in our study there was no significant difference in the occurrence of infection episodes between the soap-and-water arm and the control arm. This was due to a statistically significant decrease in the number of infection episodes in the control arm, obviously due to the national hygiene campaign. There was no concomitant decrease in the occurrence of infections in the two intervention arms. Somewhat unexpectedly, even if the infection episodes were reduced in the soap and water arm, there was no reduction in the number of sick leave or absence episodes due to infectious disease. Rather, after the onset of the pandemic, the number of episodes in the soap-and-water arm was higher than in the control arm. We speculate that people in the intervention clusters may have obediently followed the overall instructions given at the beginning of the study, including the concept that coming to work with symptoms is likely to put colleagues at risk of contracting the disease and is thus not recommended.

Our study has potential weaknesses. Firstly, we used subjective reporting of disease episodes rather than professional assessment of symptoms and signs of infection. However, we believe that the written instructions, clear definitions repeated in the weekly emails, and rapid responses to any enquiries ensured sufficiently reliable lay-person diagnosis. The simple and user-friendly web-based data collection system with a short recall-time without the need for personal home diaries contributed to high reporting coverage throughout the entire study period even in the control clusters. Secondly, we had no direct measure of individuals' adherence to the given instruction in the different intervention arms. The repeated interviews on transmission-limiting habits indicated that the overall level of implementation of the recommended measures, which was fairly high already at the base line, further improved during the study in both intervention arms and moderately also in the control arm. It is likely that the national anti-pandemic campaign had a major role in the observed "leakage" of transmission limiting behaviour to the control arm. Furthermore, already participation in an intervention trial testing the role of hand hygiene, even if in the control arm without specific instructions, is likely to affect one's behaviour based on common sense and general knowledge. Some "leakage" was therefore expected. This view is also indirectly supported by the intense interest in the study by participants in all arms, recorded by the study nurse during the monthly visits throughout the study. The third interview at the end of the study, several months after the peak in media publicity of the pandemic, suggested that some changes in behaviour among the controls had been short lived, and now the difference between the controls and the intervention arms was much clearer again. A similar finding on the post-pandemic decline in hand sanitizer use was reported from New Zealand in December 2009 following the rapid decline in media coverage of the pandemic [20]. Based on the above, we believe that the participants in our intervention arms followed the instructions fairly well.

Self-evidently a large and long-lasting intervention study among office work employees conducting their regular work requires balancing between scientific ambitions and feasibility, not forgetting costs of the study. However, given the identified limitations of this study we would have suggestions for future hand hygiene studies in order to avoid some of the problems faced in this study. Firstly, active follow up of all participants for illnesses with a mechanism to collect specimens for laboratory testing of as many illnesses as possible would enable a more precise identification of infection etiology and confirm specific effects of interventions. Secondly, it would be wise to include external objective assessments of adherence such as measurement of soap/alcohol usage in offices and observation of visits to sinks, e.g. via electronic tags.

## Conclusions

We conclude that this trial has shown that improved personal hygiene measures consisting of transmission-limiting behaviour in coughing, sneezing, and shaking hands, combined with frequent hand washing with soap and water can reduce the occurrence of self-reported acute illnesses in common office work environment. The difference to the control arm was significant even though we observed significant "leakage" of the improved behaviour to the controls and in spite of the confounding effect of the emerging influenza H1N1 pandemic during the follow up. Unlike some other studies, we did not see a rate reduction in the intervention arm that received instructions to clean hands by rubbing with an alcohol-based disinfectant.

## Competing interests

The authors declare that they have no competing interests.

## Authors' contributions

CSK participated in finalization of the study plan, in acquisition of the data, coordination of the project and analysis of the samples and data as well as drafted the manuscript. JH and PAP designed the database and participated in the study design and data analysis. TZ participated in the design of the study and coordinated the sample logistics and analysis. TK advised hand hygiene and participated in the data acquisition. AA performed the statistical analysis of the data. PA, MH, HN, PiRi and JT participated in the study design and formed connections to the participating companies. PH, MR, PeRu and EV participated in the study design. TH designed the study, participated in the coordination of the project and analysis of the data as well as in the drafting of the manuscript. All authors read and approved the final manuscript.
